# Stochasticity in the Genotype-Phenotype Map: Implications for the Robustness and Persistence of Bet-Hedging

**DOI:** 10.1534/genetics.116.193474

**Published:** 2016-10-20

**Authors:** Daniel Nichol, Mark Robertson-Tessi, Peter Jeavons, Alexander R.A. Anderson

**Affiliations:** * Department of Computer Science, University of Oxford, OX1 3QD, United Kingdom; † Department of Integrated Mathematical Oncology, H. Lee Moffitt Cancer Center and Research Institute, Tampa, Florida 33612

**Keywords:** evolution, bet-hedging, genotype–phenotype map, bacterial persistence, drug resistance

## Abstract

Nongenetic variation in phenotypes, or bet-hedging, has been observed as a driver of drug resistance in both bacterial infections and cancers. Here, we study how bet-hedging emerges in genotype–phenotype (GP) mapping through a simple interaction model: a molecular switch. We use simple chemical reaction networks to implement stochastic switches that map gene products to phenotypes, and investigate the impact of structurally distinct mappings on the evolution of phenotypic heterogeneity. Bet-hedging naturally emerges within this model, and is robust to evolutionary loss through mutations to both the expression of individual genes, and to the network itself. This robustness explains an apparent paradox of bet-hedging—why does it persist in environments where natural selection necessarily acts to remove it? The structure of the underlying molecular mechanism, itself subject to selection, can slow the evolutionary loss of bet-hedging to ensure a survival mechanism against environmental catastrophes even when they are rare. Critically, these properties, taken together, have profound implications for the use of treatment-holidays to combat bet-hedging-driven resistant disease, as the efficacy of breaks from treatment will ultimately be determined by the structure of the GP mapping.

TREATMENT resistance in many diseases is driven by the pre-existence of resistant phenotypes within the population. Why such phenotypes coexist (with sensitive phenotypes), and persist in environments never exposed to drug treatment, remains a significant unanswered question. Phenotypic heterogeneity has been observed within isogenic populations of a number of organisms, and at many scales (Balázsi *et al.* 2011), from the unicellular—bacteria (Veening *et al.* 2008), fungi (Levy *et al.* 2012), or cancer cells (Gupta *et al.* 2011)—through insects (Danforth 1999; Hopper 1999), plants (Childs *et al.* 2010), and even aspects of human development (Tonegawa 1983). Importantly, this intercellular variation has been observed even in homogeneous and constant environments, suggesting that aspects of organismal phenotype may be stochastically determined.

In environments that fluctuate unpredictably, this phenomenon can serve as a survival mechanism by increasing the likelihood that at least some offspring are well-adapted to future environments. Thus, nongenetic, nonenvironmentally-driven variation in phenotypes has been termed *bet-hedging*, as a species diversifies the phenotypes within the population in order to “hedge its bets” against environmental change [see Seger (1987) for justification of this naming, and de Jong *et al.* (2011) for a discussion of what evolutionary phenomena can be considered bet-hedging]. Oscillatory environments are common in a range of ecological settings, including fluctuating climates, immune–pathogen interactions, or cyclic hypoxia within tumors, and the range of phenotypic traits that are thought to display stochastic determination is just as broad.

Bet-hedging can offer a survival mechanism in the event of rare catastrophic environmental change. An important clinical example is that of persister cells that arise stochastically within isogenic populations of infectious bacteria such as *Escherichia coli* (Balaban *et al.* 2004; Lewis 2006; Veening *et al.* 2008). These cells, which constitute a small fraction of the population [<1%, Lewis (2006)], have reduced metabolism, and shut down nonessential cellular functions. In this dormant state, the persister cells are tolerant to the cytotoxic effects of a number of antibiotic agents. Although dormant, these cells can retain the ability to proliferate (although at a drastically reduced rate), and, when antibiotic treatment ceases, persisters will proliferate, producing nonpersisters and driving the re-emergence of the bacterial population. Hence, bet-hedging, by creating a small subpopulation impervious to those therapies that act on proliferating cells, proves to be an effective survival mechanism against antibiotic treatment. Indeed, bacterial persisters are thought to be a contributing factor to multidrug resistance in a number of diseases (Keren *et al.* 2004; Lewis 2006; Nikaido 2009), and are implicated in the dormancy of chronic diseases, such as tuberculosis, which can be suppressed but not eradicated (Zhang *et al.* 2012). Novel treatment strategies capable of effectively killing persister cells are desperately needed, and this need will continue to grow with the increasing incidence of resistance to our presently most effective antibiotics.

In cancer, bet-hedging has been minimally studied; however, a number of aspects of disease course suggest that bet-hedging mechanisms may be important for understanding how tumors evade therapy. Significant regression of tumors post-therapy leads to a period of remission, followed by the regrowth of aggressive, therapy-resistant lesions. These dynamics can be explained by the clonal model of cancer (Greaves and Maley 2012), wherein recurring drug-resistant cells are those that have stochastically acquired resistance mechanisms through genetic mutation. However, the high frequency of tumor recurrence in many cancers suggests that therapeutic escape cannot be based solely on mutational “luck.” Experimental results have shown evidence of transitory resistance (Kurata *et al.* 2004; Yano *et al.* 2005) indicative of the existence of a small drug-resistant subpopulation that re-establishes a drug-sensitive cancer cell population. Recent experiments have identified the existence of such populations of “cancer persister cells” in a cell line of EGFR+ nonsmall cell lung cancer (Sharma *et al.* 2010), indicating that bet-hedging may play a role in the emergence of cancer drug resistance (Ramirez *et al.* 2016). Thus, an understanding of bet-hedging in normal and abnormal (*e.g.*, cancer) cell function may help us understand why certain types of therapies fail while others succeed.

Müller *et al.* (2013), as well as others (Thattai and Van Oudenaarden 2004; Kussell and Leibler 2005; Wolf *et al.* 2005), have demonstrated mathematically the selective advantage of bet-hedging strategies in stochastically fluctuating environments. Showing that fitness is maximized when the probability of individuals taking certain phenotypes matches the likelihood of the environment selecting for that phenotype, provided that fluctuations are not sufficiently slow that adaptation through genetic mutation can occur, or so fast that no individuals of any phenotype can survive and reproduce. Further theoretical work by Botero *et al.* (2015) considers when bet-hedging can offer a greater fitness advantage than *phenotypic plasticity*, where phenotypes are modulated via the environmental variation (Via and Lande 1985). This previous work derives constraints on the cost of sensing, predictability of environmental fluctuations, and the fitness effects of environmental change to determine when bet-hedging, plasticity, or determinism offers a selective advantage.

It has been suggested that drug-insensitive cells that arise stochastically in an isogenic population can facilitate the emergence of genetically driven resistance by providing a window of opportunity in which resistance conferring mutations can arise Brock *et al.* (2009). However, this window is not indefinite as drug-insensitive cells will revert to a sensitive state, and likely die in the presence of a drug. Charlebois *et al.* (2011) explored this phenomenon through a mathematical model that incorporates switching from a drug-insensitive to a drug-sensitive phenotype as the stochastic relaxation from a state of high to low gene expression. This latter study demonstrated that the timescale of relaxation necessary to facilitate a high likelihood of genetic resistance is comparable to timescales measured for certain genes implicated in human cancers. In further work, Charlebois *et al.* (2014) introduced a model of a feed-forward transcriptional regulatory network to demonstrate that the network architecture can extend the time that drug-insensitive cells maintain their phenotype, and, thus, can increase the likelihood of therapeutic escape occurring through genetically driven mechanisms. This work highlights that, to understand bet-hedging-driven drug resistance, it may be necessary to look beyond the genetic scale, and toward the gene–gene interactions that comprise genotype–phenotype (GP) mapping.

The mathematical results of Müller *et al.* (2013) and others (Thattai and Van Oudenaarden 2004; Kussell and Leibler 2005; Wolf *et al.* 2005) suggest a paradox when compared to clinical observations of bet-hedging, for example, bacterial persistence, as a survival mechanism against rare catastrophic events. Specifically, in hospitable environments, bacterial persisters reproduce more slowly than cells with a proliferative phenotype, reducing population fitness. It follows that natural selection will act to remove or minimize the number of persisters in the population. Where catastrophic events are rare, we should expect bet-hedging strategies to be lost before the event occurs, resulting in extinction of the population when it eventually does. In this work, we suggest, following a similar argument to that provided by Charlebois *et al.* (2014), that the architecture of the molecular interactions networks may slow the evolutionary loss of bet-hedging to preserve it as a survival mechanism.

## The causes of bet-hedging

A number of causes of bet-hedging have been identified across different species, but in many cases the cause remains an open question. The difficulty in identifying the precise drivers lies in distinguishing between the variability (or *noise*) introduced at different biological scales. For example, gene promoter, transcription, and translation dynamics are driven by inherently stochastic molecular interactions that result in the expression of gene products that vary both temporally and between isogenic individuals (Elowitz *et al.* 2002; Kaern *et al.* 2005; Blake *et al.* 2006; Raj and van Oudenaarden 2008). These gene products interact in nonlinear, molecular networks, often forming feedback loops that have the potential to suppress (Becskei and Serrano 2000), or amplify, noise (Hasty *et al.* 2000), or induce oscillations in the concentrations of molecules (Cardelli and Csikász-Nagy 2012; Cardelli 2014). This intracellular system is further modulated by variability in environmental factors and intercellular signaling.

Current biological thought is that noise in the levels of specific intracellular proteins may drive nongenetic phenotype differentiation. Indeed, under certain regimes of promoter switching, transcription, and translation, protein abundance can follow a bimodal distribution (Kaern *et al.* 2005), inducing two distinct phenotypes in a population. Further, theoretical modeling, coupled with experimental validation, has highlighted how bistable autoregulatory genetic motifs can induce bimodal protein distributions (Hasty *et al.* 2000; Becskei *et al.* 2001; Isaacs *et al.* 2003).

The evolutionary origin of bet-hedging is unclear. It is not known whether bet-hedging emerged as an adaptation to unpredictable environments, or as a spandrel (Gould and Lewontin 1979), arising from the inherently stochastic nature of the biochemical reactions governing cellular behavior, and later co-opted as a survival mechanism. What is clear is that bet-hedging strategies, manifested as phenotypically heterogeneous populations, are subject to natural selection. Beaumont *et al.* (2009) demonstrated the *de novo* evolution of bet-hedging in the phenotypic trait of colony morphology of the bacterium *Pseudomonas fluorescens* by imposing stochastically fluctuating environments through replating. The genetic driver underpinning this switching behavior was partially elucidated by Gallie *et al.* (2015), who identified a single nucleotide change in the gene *carB* as responsible for the emergence of phenotype switching; however, identifying the precise molecular pathways through which this mutation acts to produce bet-hedging remains an open problem. Following the recent development of persister isolation techniques, a number of genetic drivers thought to contribute to bacterial persistence in *E. coli* have been identified (Lewis 2006). However, while overexpression, or deletion, of these genes were shown to impact the *proportion* of bacterial persisters within a population, none was found to completely inhibit the persister phenotype, suggesting that bet-hedging may arise from the interactions of multiple gene products.

**Figure 1:**
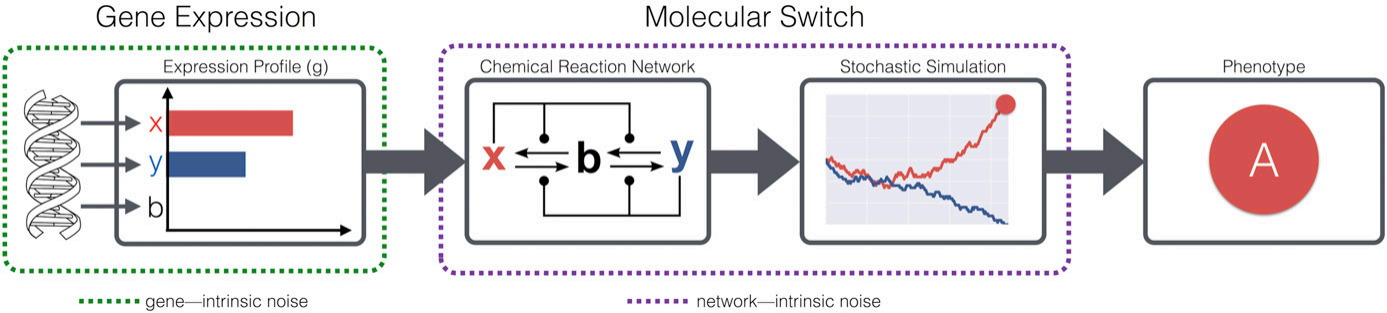
Schematic representation of the CRN model for determining phenotypes from genotypes. The gene expression profiles (*g*) are assumed to be fixed for each genotype, and the dynamics of expression from the biological genotype (green dashed leftmost box) are ignored. These modeling assumptions allow us to explore the implications of network-intrinsic noise (purple dashed centered box) independently of gene-intrinsic noise.

To address the difficulty in identifying genetic drivers of bet-hedging, we introduce a model GP mapping, wherein phenotypes emerge through the stochastic interactions of proteins in intracellular molecular pathways. Specifically, we simulate minimal interaction networks encoding bistable switches among two or three chemical species. The dynamics of these switches are implemented as chemical reaction networks (CRNs) simulated stochastically. Through this model we explore bet-hedging, which arises naturally, from the perspective of *network intrinsic noise*, as opposed to the *gene intrinsic* perspective of previous modeling (Figure 1). Under this model, stable configurations of a reaction network are analogous to local minima in an epigenetic landscape [for example, as used by Huang (2009, 2012) to study phenotypic heterogeneity in cancer].

There exist a number of regulatory motifs that induce bistable switching; however, the evolutionary implications of structural differences between these motifs have remained unexplored. Specifically, what properties of the molecular networks are likely to be selected for, and whether such properties can be exploited to identify potentially novel therapies, remain open questions. By considering simple, minimalistic models of bistable switches built from direct and indirect feedback mechanisms, we demonstrate that the structure of the networks governing phenotype differentiation can result in bet-hedging that is robust to major alterations. We argue that this robustness offers a potential explanation for the difficulty in identifying single genetic drivers of bet-hedging. Further, we demonstrate that network structure can alter the rate of evolutionary convergence to fitness optima, reducing evolvability and preventing the loss of bet-hedging in fixed environments. This result suggests a solution to the apparent paradox of bet-hedging: how can it persist for long periods of time in environments where natural selection acts to remove it? Finally, we discuss the implications of this result for the design of treatments for diseases which display nongenetic phenotypic heterogeneity.

## Materials and Methods

In this work we implement model GP mappings that stochastically determine one of two phenotypes, *A* or *B*, from a single genotype, *g*, encoding gene expression. The GP mapping can be considered to determine a genotype–dependent probability, p(g), of an individual taking phenotype, *A*. We aim to investigate the rate of evolutionary loss of bet-hedging by calculating the probability that an individual mutant invades an otherwise isogenic population. This probability is dependent on the distribution of phenotypes in the resident population. As such, this section is structured as follows. First, we derive the distribution of phenotypes, and average growth rate of a population in which each individual has probability, *p*, of having phenotype *A* at birth (and 1−p of having phenotype *B*). Second, we derive the probability of a single mutant individual with probability $p^{\prime }$ (corresponding to genotype $g^{\prime }$) invading this resident population. Third, we outline the model for the GP map, wherein p(g) is determined from *g* by a bistable CRN. Finally, we describe simulations of long-term evolution and drug therapy that form the basis of our results.

### Population dynamics

Assume a fixed GP map that determines a probability, *p*, of an individual with genotype *g* having phenotype *A* at birth. This phenotype is fixed throughout the life of the individual. As we wish to study evolutionary loss of bet-hedging, we may assume p∈(0,1), as when p=1 we will end our simulations. We also assume a fixed environment. A discrete time model is used to simulate the population dynamics as follows. Denote by $x\left(t\right)={\left[x_{A}\left(t\right),x_{B}\left(t\right)\right]}^{⊺}$ the number of individuals of phenotype *A* and *B* at discrete timestep *t*. We assume Wright-Fisher sampling, wherein each individual in the population at time *t* can contribute any number of individuals to the population at time t+1. Denote by $w_{A}$ (respectively, $w_{B}$) the expected number of individuals in the population at time t+1 that are descended from a single individual of phenotype *A* (respectively, *B*) present in the population at *t*. We assume $w_{A},w_{B}>0.$ Each new offspring takes phenotype *A* with probability *p* (and *B* with probability 1−p), where *p* is determined by the genotype, *g*, and the GP mapping.

For a fixed population size, the dynamics of the population can be modeled using mathematics from the theory of quasispecies that describe a population in mutation–selection balance (Wilke 2005). Specifically, if the average number of individuals at time t+1 produced by a single individual at time *t* is given by 〈w〉, the population dynamics are governed by the projection matrix,

$$P=\frac{1}{\langle w\rangle }\left[\begin{array}{cc} w_{A}p & w_{B}p\\ w_{A}\left(1-p\right) & w_{B}\left(1-p\right) \end{array}\right].$$ (1)

The population distribution after one discrete time step is given by x(t+1)=Px(t). As p∈(0,1), the matrix P is positive, and the Perron-Frobenius theorem (Cushing 1998; Li and Schneider 2002) tells us that the normalized eigenvector corresponding to the dominant eigenvalue of P gives the long-term stationary distribution of the two phenotypes *A* and *B*. As we have only two phenotypes, we can easily determine this dominant eigenvalue as

$$\begin{array}{c} 0=\left|P-I\lambda \right|=\left|\begin{array}{cc} \frac{w_{A}p}{\langle w\rangle }-\lambda & \frac{w_{B}p}{\langle w\rangle }\\ \frac{w_{A}\left(1-p\right)}{\langle w\rangle } & \frac{w_{B}\left(1-p\right)}{\langle w\rangle }-\lambda \end{array}\right|\\ =\left(\frac{w_{A}p}{\langle w\rangle }-\lambda \right)\left(\frac{w_{B}\left(1-p\right)}{\langle w\rangle }-\lambda \right)-\frac{w_{A}w_{B}p\left(1-p\right)}{{\langle w\rangle }^{2}}\\ =\lambda \left(\lambda -\frac{w_{A}p}{\langle w\rangle }-\frac{w_{B}\left(1-p\right)}{\langle w\rangle }\right). \end{array}$$

Thus, $\lambda =\frac{1}{\langle w\rangle }\left[pw_{A}+\left(1-p\right)w_{B}\right].$ It is easy to verify then that x∗=(p,1−p) is the normalized eigenvector corresponding to this eigenvalue as

$$P\left(\begin{array}{c} p\\ 1-p \end{array}\right)=\frac{1}{\langle w\rangle }\left[\begin{array}{c} p^{2}w_{A}+p\left(1-p\right)w_{B}\\ p\left(1-p\right)w_{A}+{\left(1-p\right)}^{2}w_{B} \end{array}\right]$$ (2)

$$=\frac{1}{\langle w\rangle }\left[pw_{A}+\left(1-p\right)w_{B}\right]\left(\begin{array}{c} p\\ 1-p \end{array}\right)$$ (3)

$$=\lambda \left(\begin{array}{c} p\\ 1-p \end{array}\right)$$ (4)

Note that this is also the distribution of phenotypes in the case where the population grows without bound (when the factor of 1/〈w〉 is omitted). From this stationary distribution, we can derive $\langle w\rangle =pw_{A}+\left(1-p\right)w_{B}.$ This phenotype equilibrium will be used in the next section to determine the invasion probability of a different (*i.e.*, mutant) bet-hedging population into an existing one.

In reality, the values $w_{A}$ and $w_{B}$ are dependent on a number of stochastic processes, most importantly reproduction and death. Later, we will consider the effect of an increased death rate associated with drug treatment, so it is informative to consider the relationship between parameters governing the explicit processes of birth and death, and the offspring numbers $w_{A}$ and $w_{B}.$ Suppose individuals of type *A* (respectively, *B*) die with probability $d_{A}$ (respectively, $d_{B}$) over each time step. Further, suppose that individuals of phenotype *A* (respectively, *B*) that survive reproduce with probability $f_{A}$ (respectively, $f_{B}$) over the timestep. In this case

$$w_{i}=\left(1-d_{i}\right)\left(1+f_{i}\right)\;\text{for}\;i\in \left\{A,B\right\}.$$ (5)

Note that multiple pairs of values for $f_{i}$ and $d_{i}$ can yield the same $w_{i}.$ The population dynamics we present here, and the invasion dynamics presented below, are identical for all such pairs.

### Invasion dynamics

Our aim is to determine the long-term evolutionary dynamics of bet-hedging populations endowed with differing GP–maps. It is intractable to determine these trajectories through explicit simulation alone. Instead we derive an analytic solution for the probability of a mutant genotype invading an existing isogenic population. Consider a large fixed-size population, and assume that mutation is sufficiently rare (explicitly that the mutation rate *μ* and population size *N* satisfy NμlogN≪1) that we may consider strong-selection weak mutation (SSWM) evolutionary dynamics (Gillespie 1983, 1984). Under these assumptions, we can assume that the population is isogenic, and that each time a new mutant appears in the population it either fixes as the new population genotype or becomes extinct before another can arise.

Suppose a single mutant of genotype $g^{\prime }$ arises in an isogenic population of genotype *g* and denote by $\pi \left(g^{\prime }\right)$, the probability that this mutant reaches fixation as the population genotype. This probability is dependent on the phenotype of this initial mutant, and is given by

$$\pi \left(g^{\prime }\right)=p\left(g^{\prime }\right)\pi \left(g^{\prime }|A\right)+\left[1-p\left(g^{\prime }\right)\right]\pi \left(g^{\prime }|B\right).$$ (6)

Denote $\pi =\left[\pi \left(g^{\prime }|A\right),\pi \left(g^{\prime }|B\right)\right]$, and suppose that the population size, *N*, is sufficiently large that we may approximate it by the limit N→∞. As we assume Wright-Fisher sampling for reproduction, the value of π can be determined from the theory of branching processes. In particular, π can be calculated numerically as the solution to the equation

$$1-\pi =e^{-P\pi }$$ (7)

where P is the matrix governing the population dynamics defined above.

**Figure 2:**
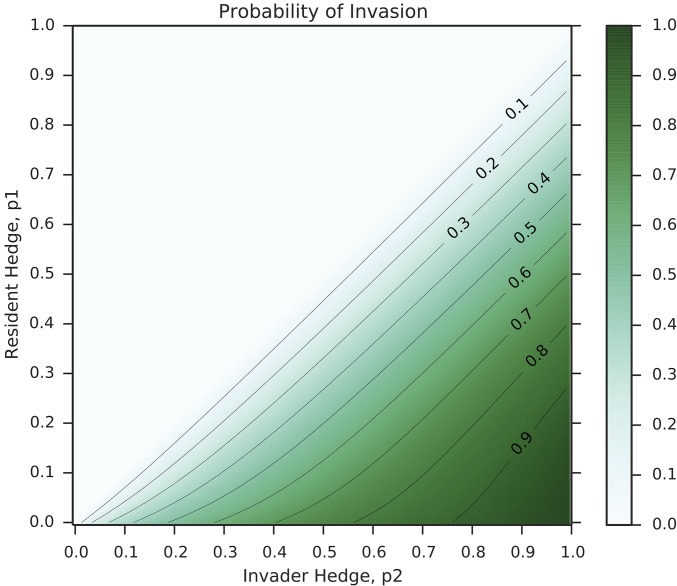
Invasion probabilities for a single mutant with genotype corresponding to a probability $p_{2}$ of producing phenotype *A* into a resident population with probability $p_{1}.$ The probabilities are calculated from Equations 6 and 7 with parameters $w_{A}=2.0,$ $w_{B}=1.01.$ Note that deleterious and neutral mutations cannot fix under our model of invasion dynamics; hence, invasion in the case $p_{2}\leq p_{1}$ (above the antidiagonal of the plot) is impossible.

A proof of this identity, modified from the theory of viral quasispecies (Wilke 2003), is presented in Supplemental Material, File S1. As the value of 〈w〉 can be calculated from the population dynamics described above, this equation can solved numerically. Figure 2 shows an example heatmap of invasion probability for an invader with genotype corresponding to probability $p_{2}$ of phenotype *A* into a resident population with probability $p_{1}.$ The parameters are $w_{A}=2.0$ and $w_{B}=1.01.$

### CRNs as a model GP map

To study the evolution of bet-hedging, we consider the genetic drivers of changes to the probability, *p*, of an individual having phenotype *A*. We implement a model GP mapping in which phenotypes emerge with proportions that are determined from the interactions of expressed gene products. In this model, the genotype, *g*, is represented in an abstracted way, as the numbers of chemical species, labeled *x* and *y*, that are present in the cell at birth. Thus, $g=\left(x_{0},y_{0}\right)\in {\mathbb{N}}^{2}.$

**Figure 3:**
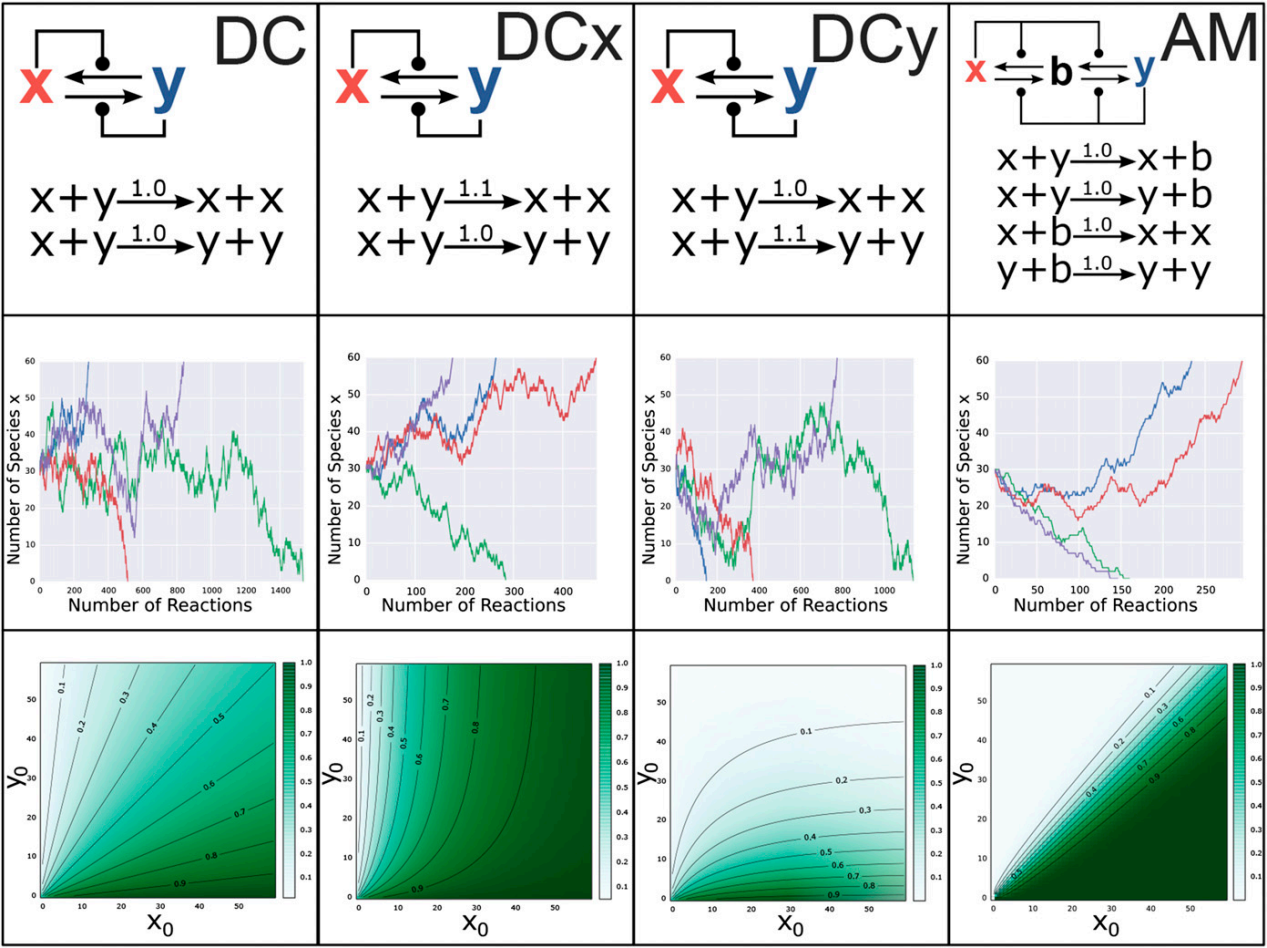
Example molecular switches as GP maps. Each column shows the characteristics of one of the four switches (DC, DCx, DCy, and AM) introduced in the main text. The first row shows the name, CRN structure, and precise definition of each switch. The second row shows stochastic trajectories of the number of molecule *x* in the system for four different simulations of each switch. The starting condition in all simulations is x=y=30, (b=0 for the AM network). Note that all of the switches are able to resolve to either of the stable conditions, x=60 or x=0, which correspond to the phenotypes *A* and *B*, respectively. Row three shows contour plots displaying the probability of switching to phenotype *A* for each possible initial condition with $0<x_{0},y_{0}\leq 60$ and $x_{0}+y_{0}\neq 0$ (b=0 for the AM switch). Contour lines show subspaces of genotype space of equal hedging probability for hedges equal to 0.1,0.2,…,0.9.

The model relies on the stochastic resolution of a CRN through the Gillespie algorithm (Gillespie 1977, 2001) to determine a phenotype from the genotype *g*. CRNs are defined by a collection of labeled chemical species and a list of reactions, with associated rates, between these species. The Gillespie algorithm determines a stochastic progression of reactions within a CRN, and returns the sequence of reactions that occur, along with the times at which they occur. We consider the class of CRNs that encode bistable switches (Cardelli and Csikász-Nagy 2012), wherein the sequence of reactions will almost surely (in the probabilistic sense) terminate in one of two stable configurations (see Figure 3). These different final configurations can be considered different states of a stochastic switch, and to represent the configurations that ultimately result in different phenotypes, *A* and *B*. The probability that the CRN progresses to a specific switch state is dependent on the initial conditions for the network. Thus, we can define a model GP mapping according to

$$g\overset{\text{GP}}{\to }\{\begin{array}{cc} A, & \text{if}\;\text{the}\;\text{simulation}\;\text{progresses}\;\text{to}\;\text{stable}\;\text{state}\;1\\ B, & \text{if}\;\text{the}\;\text{simulation}\;\text{progresses}\;\text{to}\;\text{stable}\;\text{state}\;2. \end{array}$$ (8)

A schematic representation of this model is shown in Figure 1. In this model, an individual has a phenotype that is determined soon after birth and fixed until that individual reproduces. This model of the GP map can be considered to be a stochastic, irreversible developmental program in the case of differentiating cancer cells or higher organisms. In the context of bacteria, the developmental perspective is less appropriate, and the model can be justified by considering gene expression bursts that are cell-cycle dependent (specifically, bursts that occur at the start of the G1 phase).

Note that the genotype *g* corresponding to a stable gene expression profile is the sole heritable determinant of phenotype in this model. The state of the molecular switch, *i.e.*, the phenotype, of a parent individual has no influence on the phenotype of the offspring. This assumption can be justified as follows. The bistable switches we discuss may represent only a small subnetwork of the complex and dynamic interaction network governing the GP mapping. Thus, we can expect the chemical species comprising the molecular switch, *x* and *y*, to be further transformed, or consumed, in additional unmodelled reactions that determine phenotype. Further, even when this is not the case, we can expect the *x* and *y* molecules to decay over time. Thus, the omission of epigenetic inheritance of switch state can be considered an assumption that the time scale of molecular decay is much faster than that of cellular division. Weakening this assumption, and permitting epigenetic inheritance, represents a potential extension of the model that is briefly explored in our *Discussion*.

Finally, we assume that the series of chemical reactions that result in a stable configuration for the network all occur within a sufficiently short time period (in comparison to the cell cycle) that we may take them to have all occurred instantaneously. This assumption permits us to ignore the timing information provided by the Gillespie algorithm. As such, the abscissas of all figures showing stochastic simulations of CRNs presented in this work measure time discretely, in terms of the number of reactions that have occurred, instead of continuously. The time between successive reactions in the Gillespie algorithm is dependent on a propensity function that accounts for reaction rates and the volume of the container (*i.e.*, cell cytoplasm). As we are only interested in the probability of finding each stable configuration, and not the precise time taken to reach this configuration, we may set the volume to be an arbitrary constant, say 1.0 mm^3^, and also normalize the reaction rates such that at least one reaction has rate $1.0\;{\text{sec}}^{-1}.$

Four model bistable switches that can serve as model GP maps are shown in Figure 3, along with examples of stochastic realizations of the switches (second row), and heatmaps showing $p\left[\left(x_{0},y_{0}\right)\right]=\mathbb{P}\left[g\overset{\text{GP}}{\to }A\right]$ for $x_{0},y_{0}\in \left\{0,\ldots ,60\right\}$ (third row). The value of $p\left[\left(x_{0},y_{0}\right)\right]$ is required to determine the population and invasion dynamics as described above. Estimating this value numerically through multiple samples of the Gillespie algorithm is prohibitively slow. Instead, the value can be determined analytically for the DC, DCx, and DCy switches as they correspond to the classical “drunkard’s walk” of probability theory. For the AM switch, no such analytic solution is possible. To determine $p\left[\left(x_{0},y_{0}\right)\right]$ in this case, we construct the Markov chain on the space of possible configurations of (x,y,b) and numerically solve for $p\left[\left(x_{0},y_{0}\right)\right].$ The details are provided in File S1.

### Simulating evolutionary loss of bet-hedging

To investigate the impact of GP mapping on the evolutionary loss of bet-hedging, we implement a stochastic simulation of mutation and selection. We consider mutations to a genotype $g=\left(x_{0},y_{0}\right)$ as changes to the initial abundances $x_{0}$ and $y_{0}.$ The possible mutations are thus modeled by

$$\mu \left[\left(x_{0},y_{0}\right)\right]=\left\{\left(x_{0}\pm 1,y_{0}\pm 1\right)|\;\text{provided}\;x_{0}+y_{0}=g_{\max}\right\}.$$ (9)

Note that we have restricted mutations such that total expression is conserved, and the genotype is determined entirely by the value $x_{0}\left(y_{0}=g_{\max}-x_{0}\right).$ For the remainder of this work, we omit reference to $y_{0}$, and equate *g* with $x_{0}.$ Owing to the computational complexity of our simulations, we take $g_{\max}=60.$ Changes to the value of $g_{\max}$ do not change the qualitative results, but will change them quantitatively as the time of evolutionary convergence to a nonhedging strategy increases as $g_{\max}$ is increased (see File S1).

The mutations defined by Equation 9 differ from previous network models of the GP mapping; for example, the models of Huang (2009) or Gerlee and Anderson (2009), as they modify the initial conditions of a network-defined dynamical system, as opposed to the system itself. This choice of mutation is appropriate to the level at which genotypes are modeled. For example, the phenotypes of the gene regulatory network model studied by Huang (2009) are stable gene expression profiles. By contrast, in our model, expression profiles are taken as the genotypes, the initial expression levels of *x* and *y*. This notion of genotype is chosen to allow us to investigate the impact of network-intrinsic noise on the evolution of bet-hedging (see Figure 1).

As discussed above, we assume a large asexually reproducing bet-hedging population exists under SSWM dynamics (Gillespie 1983). Our evolutionary simulation proceeds by repeatedly generating a mutant of the population genotype *g* according to Equation 9, computing the probability *π* that this mutant fixes as the new population genotype according to Equation 7, and then stochastically deciding whether the mutation fixes [by sampling q∼Unif(0,1) and comparing the value to *π*]. The simulation terminates when the genotype satisfies $x_{0}=g_{\max}$, and the total number of mutations that are sampled in the simulation (including those that do not fix) is returned as a proxy for the time until evolutionary loss of the bet-hedging. The initial population genotype, $g_{0},$ is chosen such that $p\left(g_{0}\right)\approx 0.5.$

### Simulating treatment holidays

To explore the clinical impact of treatment holidays on treating disease with bet-hedging driven resistance, we implemented a simulation comprising two parts. In the first, an evolutionary simulation similar to the one described above is performed to determine the population genotype following a treatment holiday. From an initial genotype $g_{0}$ with $p\left(g_{0}\right)\approx 0.5$, the expected population genotype *g* following a treatment holiday of length *T* (measured in mutational events) is computed. The expected time, in mutational events, taken for the genotype g+1 to arise by mutation and fix in the population is 1/[0.5π(g+1)]. Here, the factor of 0.5 arises as only half of mutations are beneficial (g→g−1 is not). Using this fact, an expected postholiday genotype can be easily determined by repeatedly incrementing *g*, while keeping a sum of the expected number of mutational events required for that new *g* to fix in the population. This process is terminated when this number of events exceeds *T*.

**Figure 4:**
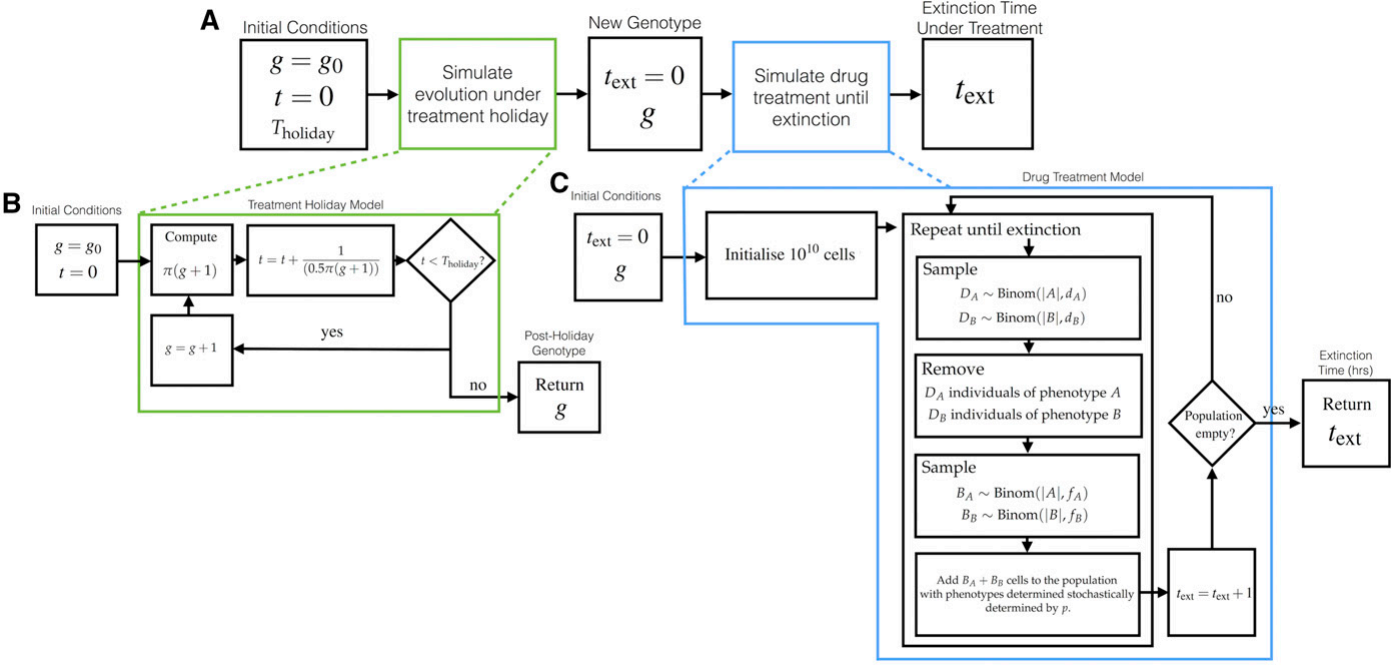
The model for simulating treatment holidays. (A) The overall model consists of first determining a post-treatment holiday genotype, *g*, and then simulating drug treatment on a population with bet-hedging determined by that genotype. (B) The expected postholiday genotype is determined from the invasion probabilities π(g), which are, in turn, dependent on the molecular switch. (C) A stochastic death–birth process is used to determine an extinction time, $t_{\mathrm{ext}}.$

Using this postholiday genotype, and the associated GP-map-dependent hedging probability p(g), a stochastic death–birth process without mutation was performed to determine an approximate time to extinction. A population of size $10^{10}$ was initialized with a proportion p(g) of phenotype *A* and [1−p(g)] of phenotype *B*. The simulation proceeds as depicted in Figure 4, and is terminated when the population is extinct, or 20,000 simulated hr has elapsed. Each time step corresponds to 1 hr. Unlike the simulation of evolutionary timescales, the treatment simulation is dependent on the parameters of per-hour probability of death, $d_{i}^{\text{drug}},$ and reproduction $f_{i}^{\text{drug}},$ for individuals of each phenotype in the drug-treated environment. This environment is different to the hospitable environment taken for evolutionary simulation.

This simulation was performed 2000 times for each treatment holiday length *T* ∈ (0, 3000, 5000, 50,000, and 100,000), and each molecular switch from Figure 3. The extinction times were collated to form the histograms shown in Figure 8.

### Data availability

All models described in this work were implemented in Python. All scripts are available from D.N. upon request.

## Results

We have implemented a model GP mapping in which phenotypes emerge with proportions that are determined from a genotype and molecular switch. It is infeasible to explicitly model the full array of chemical interactions comprising the translation from genes to phenotypes. However, investigation of smaller network motifs can provide insight into the properties of the full molecular network. A similar approach was taken by Cardelli (2014), who studied emulation between CRNs, the phenomenon where one network is capable of reproducing the exact mass-action kinetics of another. Identifying emulations provides a method to extend results gained from studying of simple CRN motifs to larger molecular pathways.

We consider four different bistable switches that can serve as model GP mappings. These switches are constructed from minimal interaction encoding direct and indirect feedback among two or three chemical species, and represent the simplest possible implementations of bistable networks. The switches, along with examples of their dynamics, are presented in Figure 3. The *Direct Competition* (DC) switch (along with DCx, DCy) consists of a pair of autocatalytic reactions. The *Approximate Majority* switch, studied by Angluin *et al.* (2008) and later Cardelli and Csikász-Nagy (2012), consists of two catalytic and two autocatalytic reactions. A biological implementation of the AM switch is presented by Dodd *et al.* (2007) as a potential mechanism for epigenetic cell memory. By picking appropriate genotypes (*i.e.*, initial conditions for the molecular network), any switching probability, and, equivalently, any ratio of phenotypes *A* and *B*, can be closely approximated using any of the switches (Figure 3, Row 3). It follows that bet-hedging can arise solely from network-intrinsic noise introduced by the stochastic interactions among as few as two chemical species. Here, we explore how the topology of simple stochastic networks influences the evolutionary fate of bet-hedging.

### Robustness and redundancy in molecular switches

**Figure 5:**
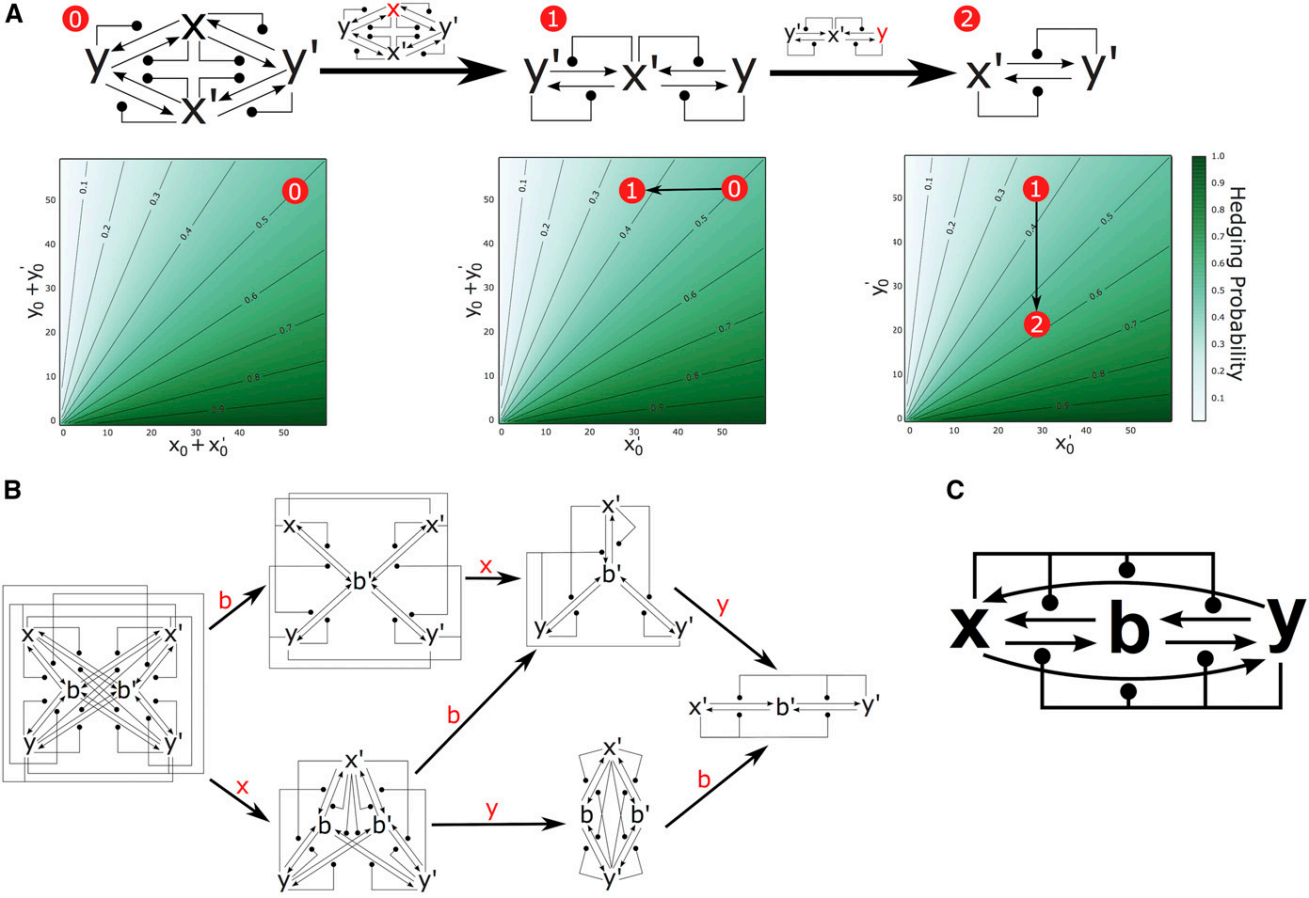
Redundancy results in bet-hedging that is robust to mutation. (A) Redundancy in the CRN implementing the DC switch maintains molecular switching when chemical species are deleted. Marked in red is the switching probability for initial conditions (20,30,30,20) before deletion (0), after the deletion of *x* (1), and after the deletion of *x* and *y* (2). Contour lines show initial conditions of equal switching behavior. (B) Redundancy in the CRN implementing the AM molecular switch. Switching is maintained if the species *x*, *y* and *b* are removed in any order. We omit the case where *y* is removed before *x* due to symmetry. (C) A molecular switch that can reduce to either AM or DC when specific reactions are inhibited.

By introducing redundancy, we demonstrate how bet-hedging can be robust to major perturbations to the underlying network. Figure 5A shows a version of the DC switch from Figure 3 in which the species *x* and *y* are duplicated. In this network, which we call DCdup, the set of stable configurations are determined by $x+x^{\prime }=0$ or $y+y^{\prime }=0.$ If we associate the phenotypes *A* and *B* with these two configurations, respectively, then the switching probability on initial conditions $\left(x_{0},x_{0}^{\prime },y_{0},y_{0}^{\prime }\right)$ is identical to the switching probability of DC with initial conditions $\left(x_{0}+x_{0}^{\prime },y_{0}+y_{0}^{\prime }\right)$ (a simple mathematical argument to establish this proceeds by symmetry and relabeling the species). The potential benefit of DCdup is that it maintains its switching properties, even if chemical species are removed. Figure 5A shows numerical solutions for the CRN switching probability when the species *x* is deleted (middle network), and then when both *x* and *y* are deleted (right hand network). These induced CRNs maintain switching behavior similar to the original network DCdup. The network induced by deleting *x* (or by symmetry *y*) behaves precisely as DCdup with initial condition $\left(x_{0},0,y_{0},y_{0}^{\prime }\right)$ [by symmetry $\left(x_{0},x_{0}^{\prime },0,y_{0}^{\prime }\right)$]. Further, removing both *x* and *y* from DCdup creates a version of the DC switch in the species $x^{\prime }$ and $y^{\prime }$ that behaves precisely as the DCdup switch on initial conditions $\left(0,x_{0}^{\prime },0,y_{0}^{\prime }\right).$ It follows that deletion of chemical species will change the likelihood of switching (and thus the proportion of phenotypes *A* and *B*) if initial numbers of all other proteins remain fixed. An example is shown by the red circles in Figure 5A, where deletion of chemical species shifts the switching probability, but does not inhibit bet-hedging entirely. Hence, the DCdup switch is robust to the removal of chemical species—a mutational event that, in our model, can be interpreted as deletion or downregulation of a gene. Reversing this argument, the switching behavior of the CRNs in Figure 5A demonstrate how bet-hedging is robust to gene duplications or upregulating mutations.

A similar redundant implementation of the AM molecular switch is shown in Figure 5B, and is robust to the removal of species, *x*, *y*, and *b* in any order. As with the DCdup network, the removal of any chemical species will change the switching probability, and shift the proportions of phenotypes in the population, but will not inhibit one phenotype entirely. Further, we note that the switching is not only robust to mutations that remove chemical species, but also to alterations in the rates of reaction between them. For example, each of the switches presented in Figure 3 can be derived from the larger network shown in Figure 5C by inhibition of specific reactions. Removal of the autocatalytic reactions x+y→x+x and x+y→y+y yields the AM switch. Alternatively, removing the four reactions involving the chemical species *b* yields the DC switch.

### Evolutionary loss of bet–hedging

For fixed gene expression, the specific switching mechanism responsible for producing multiple phenotypes is irrelevant, as the proportion of phenotypes remains fixed. However, over longer timescales, the structure of the molecular switch has a significant impact on the evolution of bet-hedging. Throughout the remainder of this work, we take the two phenotypes *A* and *B* to correspond to a high fitness, proliferative phenotype, and a low fitness, slow proliferating phenotype, respectively, mirroring the phenomenon of bacterial persistence.

The invasion probability is computed independently of the (assumed to be large) population size, and is dependent only on the (stable) distribution of phenotypes in the population at equilibrium. This distribution can be computed from the values $w_{A}$ and $w_{B}.$ We parametrize the model determined as follows. The discrete timesteps are taken to be t=60mins, mirroring an expected division time of *E. coli*. The time from birth until reproduction for a phenotype *A* individual is distributed exponentially, with rate parameter $\lambda_{A}=1.0$ per 60 min. The number of reproductions of a single individual is then Poisson distributed with parameter $\lambda_{A}=1.0.$ Hence, on average, each phenotype *A* individual present at time *t* reproduces once in the 60 min, and produces two individuals at time t+1 (itself and its offspring). It follows, $w_{A}=2.0.$ The reproductive rate of persister-type cells is unknown. To match their behavior qualitatively, we take our persister-like phenotype *B* cells to have an expected reproduction time 100 times slower than phenotype *A* individuals, at 6000 min. Thus, reproduction time for phenotype *B* cells is distributed exponentially with rate parameter $\lambda_{b}=0.01$ per 60 min. The expected number of reproductions over the 60 min timestep is then 0.01 and $w_{B}=1.01.$ The environment remains fixed ($w_{A},w_{B}$ are unchanged) throughout the evolutionary simulation. Although this parametrization is only an approximation, it is sufficient as an illustrative model demonstrating the importance of the structural properties of GP mapping. The effects of changing $w_{A}$ and $w_{B}$ are discussed in File S1.

**Figure 6:**
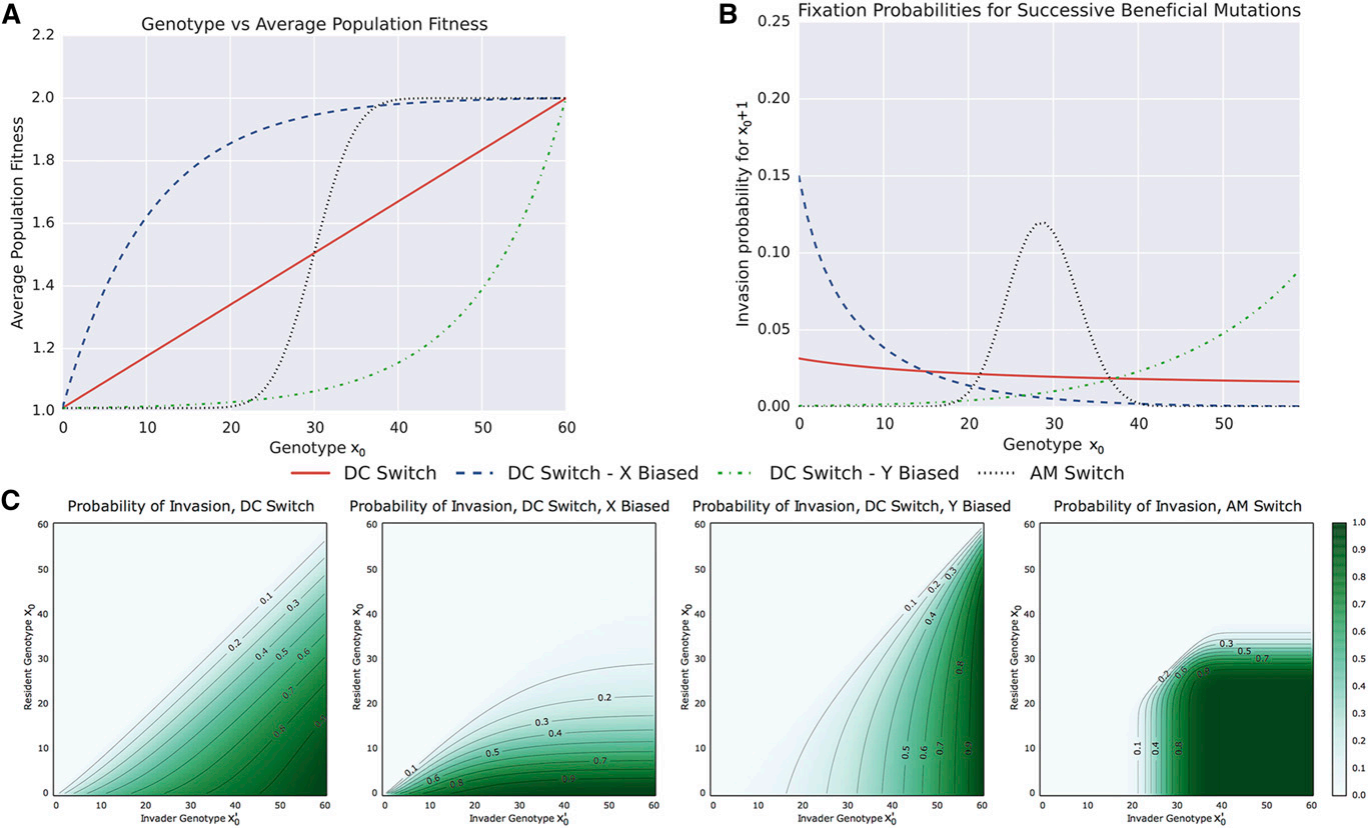
GP mapping determines the dynamics of invasion. (A) The relationship between population genotype, $x_{0},$ and average population fitness for each molecular switch. (B) Invasion probabilities for a new genotype $x_{0}+1$ into a resident population of genotype $x_{0}.$ (C) Invasion probabilities for resident and invader genotypes.

Figure 6A shows how changes in the population genotype manifest themselves as changes in the average population fitness. The expected population fitness increase associated with a mutation from $x_{0}$ to $x_{0}+1$ is not constant, and instead is dependent on the underlying molecular switch. As a result, invasion probabilities are dependent on both the population genotype, invading genotype, and the form of the molecular network. Figure 6C shows the probability of a single mutant genotype, $x_{0}^{\prime }$, invading a resident population of genotype $x_{0}.$ Note that only mutations that increase the proportion of phenotype *A* are beneficial, and, hence, as our invasion probabilities are determined from the theory of branching processes, are the only mutations that can fix. Figure 6B shows the probability of successive beneficial mutations, $x_{0}+1,$ invading a resident population of genotype $x_{0}.$ In this figure, we see the impact of the GP map on the evolutionary dynamics. For the DC, DCx, and AM switches, the probability of the next beneficial mutation fixing reduces for each successive mutation. The magnitude of this decrease is dependent on the switch, and, in the case of DCx and AM, approaches zero. Conversely, for the DCy switch, each successive mutation is more likely to fix.

**Figure 7:**
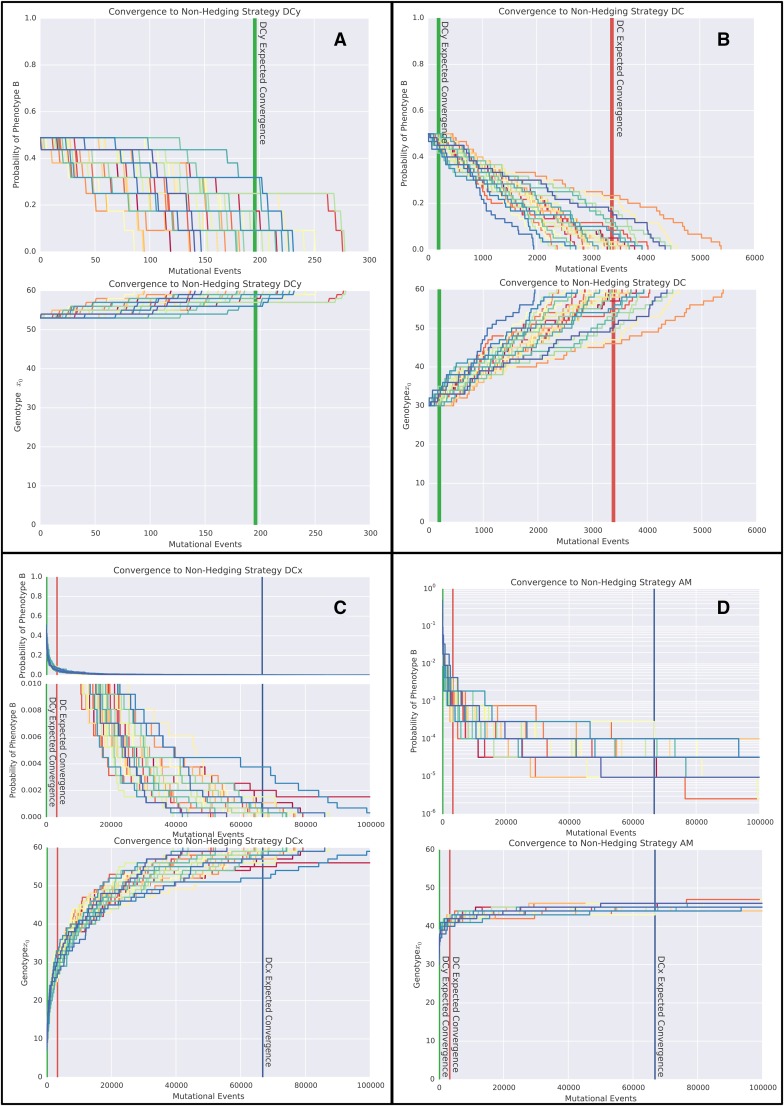
Convergence dynamics through genotype and probability space for the GP maps defined by DC, DCx, DCy, and AM; 30 stochastic realizations of the evolutionary simulation through both genotype and probability space are shown for (A) the DCy switch, (B) the DC switch, (C) the DCx switch, and (D) the AM switch. Due to the rapid initial change in hedging probability for the DCx switch, the convergence dynamics are also shown on a restricted scale. As the probability of phenotype *B* rapidly approaches zero in the AM switch simulation but never converges, the dynamics are shown on a logarithmic scale. The expected convergence time for the DCy switch is marked in green, for the DC switch is marked in red, and for the DCx switch is marked in blue.

Consider the evolutionary trajectories of phenotype proportions determined by each of the molecular switches from an initial gene expression profile corresponding closely to a population consisting of ∼50% of each phenotype. As the DC and AM switches are symmetric, the genotype corresponding to a 0.5 hedging probability is $x_{0}=30.$ For DCx, the closest genotype to a 50% hedge is $x_{0}=7$, which corresponds to a probability of 0.49. For DCy, the closest genotype to a 0.5 hedging probability is $x_{0}=53$, and corresponds to a probability of 0.51. As deleterious and neutral mutations cannot fix under our model of population dynamics, the population genotype will be periodically incremented until $x_{0}=60$, at which point the bet-hedge is lost. Figure 7 shows the evolutionary trajectories toward this loss of bet-hedging, highlighting considerably different convergence dynamics. For the DC, DCx, and DCy switches, the expected convergence times can be determined as the expectation of a sum of nonidentical independent geometric distributions. We find that the expected number of mutational events required for a complete loss of bet-hedging, as highlighted in Figure 7, are given by

$$\begin{array}{l} E\left[\text{time}\;\text{to}\;\text{loss}\;\text{of}\;\text{bet-hedge}|\text{DCy}\;\text{switch},x_{0}=53\right]=195.8\\ E\left[\text{time}\;\text{to}\;\text{loss}\;\text{of}\;\text{bet-hedge}|\text{DC}\;\text{switch},x_{0}=30\right]=3382.8\\ E\left[\text{time}\;\text{to}\;\text{loss}\;\text{of}\;\text{bet-hedge}|\text{DCx}\;\text{switch},x_{0}=7\right]=68,000.1\\ E\left[\text{time}\;\text{to}\;\text{loss}\;\text{of}\;\text{bet-hedge}|\text{AM}\;\text{switch},x_{0}=30\right]=\infty \end{array}$$

In the case of the AM network, each subsequent mutation provides a diminishing increase in fitness until mutations are approximately neutral. The probability of neutral mutations fixing within our model of invasion dynamics, which models the population size as tending to infinity, is zero. In reality, the actual convergence times in the AM will depend on the population size. For large populations, as is our assumption, the timescales will be sufficiently long that we take it as equivalent to the evolutionary trajectory never converging. (This assumption can be justified by the observation that over these time scales either unmodelled mutations [such as mutations to the GP map itself, to other genes governing the phenotypes *A* and *B*, or to other aspects of the phenotype], or unmodelled changes in the environment or ecosystem, will occur, rendering our model unsuited to the situation.)

### Simulation of therapeutic intervention

To demonstrate the importance of the underlying molecular switch on the efficacy of treatment holidays for diseases with bet-hedging-driven resistance, we performed a two-part simulation. First, a treatment holiday was simulated through an evolutionary simulation in a hospitable environment to determine a postholiday population genotype. A nonspatial, individual-based model was then used to simulate drug treatment. The parameter values used in the evolutionary simulation of treatment holidays were chosen to coincide with the values $w=\left[w_{A}^{\text{hosp}},w_{B}^{\text{hosp}}\right]=\left[2.0,1.01\right]$ used in our simulation of evolutionary loss of bet-hedging. For the simulation of treatment, explicit values of $f_{i}^{\text{drug}}$ and $d_{i}^{\text{drug}}$ (for i∈{A,B}) are required. First, we determined the values for the nondrug environment that correspond to those used in the evolutionary simulation. For the hospitable environment, we assumed a fixed death probability for both *A*s and *B*s of $d_{A}^{\text{hosp}}=d_{B}^{\text{hosp}}=0.005.$ The per-hour likelihood of reproduction for the surviving individuals was taken as $f_{A}^{\text{hosp}}=1.0$ and $f_{B}^{\text{hosp}}=0.015.$ These parameters correspond to overall expected offspring for each phenotype

$$\begin{array}{c} \left[w_{A}^{\text{hosp}},w_{B}^{\text{hosp}}\right]=\left[\left(1-d_{A}^{\text{hosp}}\right)\left(1+f_{A}^{\text{hosp}}\right),\left(1-d_{B}^{\text{hosp}}\right)\left(1+f_{B}^{\text{hosp}}\right)\right]\\ \approx \left[2.0,1.01\right]. \end{array}$$

We consider three regimes of drug–environment parameter sets corresponding to a purely cytostatic drug, affecting only $f_{A},$ a purely cytotoxic drug, affecting only $d_{A}$, and a drug that is a mixture of the two, affecting both $f_{A}$ and $d_{A}$ intermediately. In the following discussion the “drug” superscript is omitted for readability.

For each regime we assume that the parameters for phenotype *B*, mirroring a drug-impervious persister-like phenotype, are unchanged. Consider a fixed drug parameter set. For each switching network, the expected postholiday population genotype, *g*, was calculated for each treatment holiday length *T* ∈ (0, 3000, 5000, 50,000, 10,000) from initial genotype $g_{0}$ chosen, as before, to correspond closely to an equal proportion of each phenotype. From each *g*, we then performed 2000 simulations of drug treatment to determine a distribution of extinction times. Where simulation time exceeded 20,000 hr, the simulation was halted and extinction was determined to not occur. A standard course of antibiotic treatment lasts between 7 and 10 days. Using this period as a guide, we defined extinction times of <240 hr as corresponding to a potentially successful course of therapy, and thus, a viable treatment holiday strategy. The extinction time histograms lying within this period are shaded green and marked by a * in the histograms. Mutations were not modeled during the simulations of therapy.

The cytostatic drug parameter set was taken to correspond to a substantial reduction in the proliferative rate of phenotype *A* individuals. We note that extinction will almost surely never occur if

$$\begin{array}{c} \langle w^{\text{drug}}\rangle =p\left(g\right)w_{A}^{\text{drug}}+\left[1-p\left(g\right)\right]w_{B}^{\text{drug}}\\ =p\left(g\right)\left(1-d_{A}\right)\left(1+f_{A}\right)+\left[1-p\left(g\right)\right]\left(1-d_{B}\right)\left(1+f_{B}\right)\\ \geq 1. \end{array}$$

As $\left(1-d_{B}\right)\left(1+f_{B}\right)>1.0,$ it follows that a cytostatic drug can only drive extinction in this case if $\left(1-d_{A}\right)\left(1+f_{A}\right)<1.0.$ As $d_{A}=0.005,$ this occurs only if $f_{A}<0.995^{-1}-1\approx 0.005.$ For our simulation of cytostatic drug treatment, we assume that the drug is entirely effective at inhibiting reproduction, $f_{A}=0.$ For this parameter, we find that extinction never occurs within the 240 hr time period, indicating that, regardless of the underlying switching mechanism, no length of treatment holiday will result in complete cure with a cytostatic drug. The histograms of extinction times, where extinction does occur, are presented in Figure 3 of File S1.

**Figure 8:**
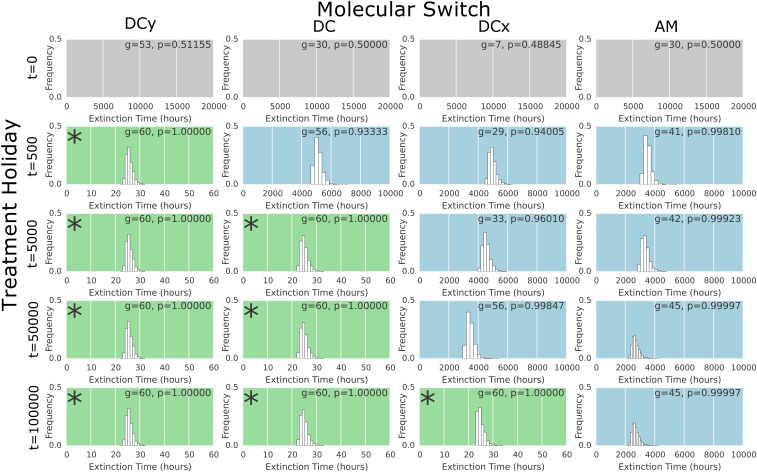
Treatment dynamics for populations endowed with the different switching networks after differing timescales of treatment holidays for a cytotoxic drug regime. Each histogram shows the distribution of extinction times over 2000 simulations of treatment in an individual-based model. The switch used as the GP map is shown as the column heading. The genotype and associated probability of phenotype *A* (shown inset to each subfigure) are determined by an evolutionary simulation of a treatment holiday for a timescale, measured in mutational events, determined by the row. The gray background (top row) indicates that no extinction occurred within the simulated 20,000 hr of treatment. The blue background indicates extinction times longer than a timeframe viable for an antibiotic treatment (240 hr), a green background (or inset star) indicates extinction times within this time frame.

For the cytotoxic drug parameter set, we consider an increased death probability of $d_{A}=0.8,$ holding all others the same. The histograms of extinction times are presented in Figure 8. Here, we see that the length of treatment holiday necessary for a successful (<240 hr) follow up course of therapy varies by orders of magnitude depending on the underlying switch. It follows that the efficacy of treatment holidays as a potential therapeutic intervention for a disease with bet-hedging-driven resistance is dependent on the underlying driver of bet-hedging.

To test the robustness of this result, we considered an intermediate parameter set of $d_{A}=0.4$ and $f_{A}=0.5$ as a trade-off between cytostasis and cytotoxicity. The histograms of extinction times in this simulation are presented in Figure 4 of File S1. We find that, although the specific extinction times change, the qualitative results remain the same. Specifically, those combinations of treatment holiday, *T*, and switching mechanism that resulted in an effective follow-up treatment with a cytotoxic drug (Green, Figure 8) also permit effective treatment under this mixed parameter set. The converse is also true, with the blue (extinction but not within 240 hr) and gray (no extinction with 20,000 hr) histograms from Figure 8 being preserved.

## Discussion

We have introduced a model for the GP map that uses minimal networks of stochastic interactions to determine phenotypes. Other models of nongenetic phenotypic heterogeneity have utilized deterministic interaction networks, for example, the models of Gerlee and Anderson (2007) or Huang (2009, 2012), or stochastic simulation of empirically derived molecular pathways, for example Charlebois *et al.* (2014). The model presented here differs as we investigate minimal stochastic instances of switching behavior. These networks, like those investigated by Gerlee and Anderson or Huang, are, at present, hypothetical. However, this work could be readily extended to empirically observed reaction networks in future work.

Remarkably simple network motifs have been demonstrated to implement switching behavior that can produce populations in which different proportions of cells, determined by the initial conditions of the network, take on different phenotypes. While abstract in its representation of reactions within a network, the reactions in our model are closely related to the physical mechanisms that govern intracellular regulatory networks, providing valuable insight into the impact of network architecture on the stochastic determination of phenotypes.

We have demonstrated how redundancy, a common feature of many biological systems, can result in bet-hedging that is robust to the addition or removal of chemical species. This redundancy, which can arise initially through neutral or nearly-neutral mutations of network structure, can ensure that bet-hedging is not lost through gene deletions or duplications. Critically, this observation may explain the failure to identify genes responsible for bacterial persistence. For example, Lewis (2006) highlights mutations to the genes *hipA*, *rmf*, *sulA*, and toxin–antitoxin (TA) loci *relBE*, *dinJ* and *mazEF* as possible drivers of bacterial persistence. However, deletion of *rmf*, *relBE* or *mazEF* has been demonstrated to have no effect on the phenomenon of persistence, owing possibly to redundancy in TA modules, while deletion or over expression of *hipA* can change the proportion of bacterial persisters but not eradicate them. This is consistent with our results that indicate deletion of a single species in the CRN will not inhibit phenotypic heterogeneity but may alter phenotype proportions (Figure 5). The conclusion to be taken from the results reviewed by Lewis (2006) need not be that the factors identified are not the ones driving bacterial persistence, but instead it may be that the search for a single genetic factor responsible for bet-hedging is doomed to fail. It may be that bet-hedging emerges from the interactions of a collection of genetic factors in the sense of the epigenetic landscape studied by Brock *et al.* (2009) and Huang (2009). If this is the case, then to identify the biological mechanisms responsible for bet-hedging, we need to move beyond a gene-centric perspective, and to identify those networks of interactions governing the determination of phenotypes.

Mutations in cancer have often been associated with their direct effect on phenotypes—the concept of a driver mutation being that it induces a novel adaptive phenotype leading to clonal expansion (McFarland *et al.* 2014). However, our results suggest another phenomenon, wherein mutations do not induce novel phenotypes, but rather alter frequencies of pre-existing phenotypes within the population. This change in phenotypic ratio can have implications for cancer progression—a phenomenon previously explored by Charlebois *et al.* (2011). Consider the phenomenon of tumorigenic cells, where it is thought that only cells of a certain phenotype can form a growing tumor mass (Pardal *et al.* 2003; Ricci-Vitiani *et al.* 2007; Meacham and Morrison 2013). Genetic heterogeneity can explain the existence of a tumorigenic subpopulation if certain driver mutations are responsible for the tumorigenic phenotype. However, an alternative mechanism is that stem-like tumor cells give rise to a population of heterogeneous phenotypes. In the traditional stem cell model, a hierarchy exists where the stem cells produce a range of tumor cell phenotypes (Reya *et al.* 2001). Cancer stem cells divide to produce either more cancer stem cells (self-renewal) or cells with nonstem phenotypes down the hierarchy. This cellular decision is often taken to be stochastic (an example of bet-hedging), and our results highlight the potential for mutations to alter the probabilities of self-renewal or differentiation that have been shown to have significant impact on many aspects of tumor progression (Enderling *et al.* 2013).

An alternative bet-hedging mechanism for tumorigenicitiy is that the tumorigenic phenotype is transient and stochastically determined (potentially with microenvironmental influence). Evidence for this phenomenon was highlighted by Quintana *et al.* (2010), who demonstrated that the tumorigenic potential of individual melanoma cells is similar, despite high heterogeneity of many markers in the initializing cell. No driver or stem population was found, and, indeed, the heterogeneity of marker expression was recapitulated by most tumorigenic cells, regardless of the starting pattern of expression. Such a mechanism would have different implications from the stem cell model presented above, as potentially sensitive cells would be more difficult to define. However, our predictions remain the same: that genetic mutations could shift the frequency of tumorigenic phenotypes and profoundly impact cancer progression.

Our results further demonstrate that the structure of the network governing phenotypic differentiation also has important implications for the evolutionary loss of bet-hedging. By considering mutations to the expression levels of genes, we find that the time taken for a two-phenotype bet-hedge to be lost, in an environment favorable to only one of the phenotypes, can vary by orders of magnitude depending on the network structure. If bet-hedging serves as a survival mechanism in the event of rare catastrophic environmental change (*e.g.*, drug treatment), then the GP mapping can prevent loss of this survival mechanism over the long timescales in which catastrophe does not occur. For example, if bet-hedging is driven by a network such as the AM network (Figure 3), then each successive mutation toward a one–phenotype strategy induces a diminishing increase in the probability of generating that phenotype, and, thus, a diminishing increase in expected fitness. Eventually mutations become (essentially) neutral, and unable to fix in a large population. It is the structure of the molecular switch that substantially slows evolutionary convergence. This result provides a possible solution to the apparent paradox of bet-hedging, *i.e.*, how can bet-hedging persist in environments where natural selection acts to remove it? The structure of the molecular mechanism, itself subject to natural selection, can slow the loss of bet-hedging strategies to ensure a survival mechanism, even where environmental catastrophes are very rare.

These results have important implications for therapeutic strategies to treat diseases displaying bet-hedging-driven drug resistance. Theoretical strategies to combat bet-hedging-induced drug resistance have focused on identifying novel agents capable of killing persister cells, or identifying genetic mechanisms that can be targeted to prevent the persister phenotype from emerging. This latter strategy bears a striking resemblance to the targeted therapy revolution in the treatment of many cancers. The identification of molecular targets whose inhibition induces death (or inhibits growth) has led to the discovery of a number of novel therapies for melanoma, nonsmall cell lung cancer, and colorectal cancers. These drugs are, in the short term, remarkably effective; however, the effects are rarely durable. Mutations that abrogate the effects of targeted therapies quickly emerge during treatment, driving resistance, and, ultimately, mortality. The results of our chemical reaction model shed light on this Darwinian adaptation, and suggest that targeted therapies to prevent bet-hedging may either be impossible, or, where they do exist, prone to fail due to the re-emergence of bet-hedging through evolution. More precisely, the discovery of a single “magic bullet” genetic factor (Strebhardt and Ullrich 2008), which, when targeted, can switch off multi-drug resistant dormant phenotypes, is unlikely, owing to redundancy in the network architecture. However, we should not rule out the potential of targeted therapies entirely. It may be possible to identify multiple targets for which simultaneous inhibition prevents bet-hedging. Alternatively, targets may be identified that shift the proportion of resistant or dormant individuals within a population to a manageable level, either permitting treatment with other cytotoxic agents, or driving the disease into a dormant state.

A second theoretical treatment strategy suggested to combat resistance in cancers and resistant infections (Bigger 1944) is the introduction of treatment holidays. The traditional doctrine for therapy is one of *maximal dose-density*, *i.e.*, that we should treat diseases using the most potent drug with the highest tolerable dose for the longest possible time until the disease is cured, the therapy ceases to be effective, or the drugs become too toxic. Mathematical models of disease progression assuming genetically driven resistance indicate that this approach could drive the emergence of resistance through an ecological principle called *competitive release* (Alto *et al.* 2013; Adkins and Shabbir 2014). Before treatment, cells compete with one another for limited resources within a spatially constrained population. In a nontreated environment, pre-existing resistant cells are often less fit than sensitive ones, and, thus, owing to competition, do not grow to large numbers within the population. Selective pressures, for example within a growing tumor, are often not sufficiently strong for clonal sweeps (the fixation of a single genetic clone) to occur (Robertson-Tessi and Anderson 2015; Sottoriva *et al.* 2015) and the population contains a heterogeneous mix of phenotypes. When this population is exposed to a maximum dose-density therapy, the sensitive cells are killed, allowing the rapid outgrowth of the previously small and resistant population. This population then drives the recurrence of drug-resistant disease. Treatment holidays have been suggested as a potential therapeutic strategy to avoid drug resistance driven by competitive release (Enriquez-Navas *et al.* 2016).

Here, we implemented an individual-based model of the dynamics of a bet-hedging population under treatment to explore the efficacy of treatment holidays in combating bet-hedging-driven resistance. Coupled with a long-term evolutionary simulation, we explored the impact of the mechanism driving bet-hedging on the efficacy of treatment holidays. Our model suggests that it is the GP map, and, in particular, how it hinders or promotes the rate of evolution, that determines the efficacy of treatment breaks in reversing drug resistance. If the mechanism driving bet-hedging permits evolutionary loss in a short-to-medium timescale, then treatment holidays may drive the loss of bet-hedging and re-establish drug sensitivity. However, if instead, the driving mechanism slows the evolutionary loss of bet-hedging, then a treatment break is unlikely to re-establish drug sensitivity in a time frame relevant to disease progression. We note that interfering with the mechanism driving bet-hedging through targeted therapy could alter the switching to allow the fast evolutionary loss of bet-hedging where previously this would not occur.

In this paper, we have taken the initial conditions of our reaction networks to be genetically determined and stable, allowing us to explore the implications of the structure of the network on the evolution of bet-hedging. The model we present could also be used to study additional aspects of nongenetic heterogeneity other than those presented here. For example, the model could be extended to directly account for the dynamics of gene expression [for example using the model presented by Kaern *et al.* (2005) or the GRN model presented by Huang (2009)]. We have assumed that the chemical species comprising the reaction networks are entirely depleted before reproduction and determination of the next phenotype. Omitting this assumption to permit the epigenetic inheritance of molecular switch state represents an extension of our model to account for phenotypic memory or carry-over. Alternatively, the initial conditions could be taken to be environmentally determined, and the network itself to be genetically determined. In this case the model would give a GP map similar to the neural network model used by Gerlee and Anderson (2007) and Gerlee *et al.* (2015) to study phenotypic plasticity. However, our model would differ in that the determination of phenotypes would be stochastic, permitting the study of environment-dependent bet-hedging strategies.

Finally, it is worth noting that the model of Gerlee and Anderson (2007) and Gerlee *et al.* (2015) is an extension of the classical concept of the reaction norm (Via and Lande 1985) to nonlinear and higher dimensional functions. The model presented here offers a further natural extension, breaking down the assumption of functionality within the reaction norm by introducing stochasticity, bringing the reaction norm concept more closely in line with biological reality. This theoretical extension of GP mapping to a stochastic nonfunctional process could be further extended to account for environmental factors, as discussed above. Such an extension will bring the model more closely in line with the maxim of developmental biology, that both environment and genotype are equally important in determining the phenotype (“*g* + *e* = *p*”). Pigliucci (2010) suggests, in a review of theoretical models of the GP mapping, that we must build models that take both environment and genotype as equal partners in determining the phenotype, and attempt to bridge the divide between developmental biology and the modern evolutionary synthesis. The model presented here represents a potential step toward this goal, but importantly offers something more than previous models that have set out along this path: an attempt to account for the role of chance.

## 
